# Height, selected genetic markers and prostate cancer risk: results from the PRACTICAL consortium

**DOI:** 10.1038/bjc.2017.231

**Published:** 2017-08-01

**Authors:** Artitaya Lophatananon, Sarah Stewart-Brown, Zsofia Kote-Jarai, Ali Amin Al Olama, Sara Benlloch Garcia, David E Neal, Freddie C Hamdy, Jenny L Donovan, Graham G Giles, Liesel M Fitzgerald, Melissa C Southey, Paul Pharoah, Nora Pashayan, Henrik Gronberg, Fredrik Wiklund, Markus Aly, Janet L Stanford, Hermann Brenner, Aida K Dieffenbach, Volker Arndt, Jong Y Park, Hui-Yi Lin, Thomas Sellers, Chavdar Slavov, Radka Kaneva, Vanio Mitev, Jyotsna Batra, Amanda Spurdle, Judith A Clements, APCB BioResource, The PRACTICAL consortium, Johanna Schleutker, BØrge G Nordestgaard, Fredrik Wiklund, Ruth C Travis, Christopher A Haiman, Stephen N Thibodeau, Christiane Maier, Vogel Walther, William J Blot, Adam S Kibel, Cezary Cybulski, Lisa Cannon-Albright, Hardev Pandha, Manuel R Teixeira, Margaret Cook, Koveela Govindasami, Michelle Guy, Daniel Leongamornlert, Emma J Sawyer, Rosemary Wilkinson, Angela Morgan, Cyril Fisher, Edward J Saunders, Malgorzata Tymrakiewicz, Naomi Livni, Steve Hazel, Tokhir Dadaev, Angela Cox, Anne George, Athene Lane, Gemma Marsden, Michael Davis, Paul Brown, John Pedersen, John L Hopper, Ami Karlsson, Carin Cavalli-Bjoerkman, Jan Adolfson, Jan-Erik Johansson, Michael Broms, Paer Stattin, Suzanne Kolb, Christa Stegmaier, Babu Zachariah, Hyun Park, James Haley, Julio Pow-Sang, Maria Rincon, Selina Radlein, Aleksandrina Vlahova, Atanaska Mitkova, Darina Kachakova, Elenko Popov, Svetlana Christova, Tihomir Dikov, Allison Eckert, Angus Collins, Glenn Wood, Greg Malone, Kimberly Alexander, Kris Kerr, Mary-Anne Kedda, Megan Turner, Pamela Saunders, Peter Heathcote, Srilakshmi Srinivasan, Tracy Omara, Trina Yeadon, Felicity Lose, Douglas Easton, Rosalind A Eeles, Kenneth Muir

**Affiliations:** 1 Centre of Epidemiology, Division of Population Health, Health Services Research and Primary Care, School of Health Sciences, Medicine and Health, The University of Manchester, Manchester M13 9PL, UK; 2 Division of Health sciences, Warwick Medical School, University of Warwick, Coventry CV4 7AL, UK; 3 Division of Genetics and Epidemiology, The Institute of Cancer Research, London SW7 3RP, UK; 4 Centre for Cancer Genetic Epidemiology, Department of Public Health and Primary Care, University of Cambridge, Strangeways Research Laboratory, Cambridge CB1 8RN, UK; 5 Nuffield Department of Surgical Sciences, John Radcliffe Hospital, Headington, Oxford OX3 9DU, UK; 6 Cancer Research UK Cambridge Research Institute, Li Ka Shing Centre, Cambridge CB2 0RE, UK; 7 Nuffield Department of Surgical Sciences John Radcliffe Hospital, University of Oxford, Oxford OX3 9DU, UK; 8 School of Social and Community Medicine, University of Bristol, Canynge Hall, 39 Whatley Road, Bristol BS8 2PS, UK; 9 Cancer Epidemiology Centre, The Cancer Council Victoria, 615 St Kilda Road, Melbourne, Victoria 3004, Australia; 10 Centre for Epidemiology and Biostatistics, Melbourne School of Population and Global Health, The University of Melbourne, Victoria 3010, Australia; 11 Genetic Epidemiology Laboratory, Department of Pathology, The University of Melbourne, Grattan Street, Parkville, Victoria 3010, Australia; 12 Centre for Cancer Genetic Epidemiology, Department of Oncology, University of Cambridge, Strangeways Laboratory, Worts Causeway, Cambridge CB1 8RN, UK; 13 Department of Applied Health Research, University College London, 1-19 Torrington Place, London WC1E 7HB, UK; 14 Department of Medical Epidemiology and Biostatistics, Karolinska Institute, Stockholm 10435, Sweden; 15 Department of Clinical Sciences at Danderyds Hospital, Stockholm 17177, Sweden; 16 Division of Public Health Sciences, Fred Hutchinson Cancer Research Center, Seattle, WA 98109-1024, USA; 17 Department of Epidemiology, School of Public Health, University of Washington, Seattle, WA 98195, USA; 18 Division of Clinical Epidemiology and Aging Research, German Cancer Research Center (DKFZ), Heidelberg 69120, Germany; 19 Division of Preventive Oncology, German Cancer Research Center (DKFZ), Heidelberg 69120, Germany; 20 German Cancer Consortium (DKTK), Heidelberg 69120, Germany; 21 Department of Cancer Epidemiology, Moffitt Cancer Center, 12902 Magnolia Drive, Tampa, FL 33612, USA; 22 Biostatistics Program, Moffitt Cancer Center, 12902 Magnolia Drive, Tampa, FL 33612, USA; 23 Department of Urology and Alexandrovska University Hospital, Medical University, Sofia 1431, Bulgaria; 24 Department of Medical Chemistry and Biochemistry, Molecular Medicine Center, Medical University, Sofia, 2 Zdrave Str., Sofia 1431, Bulgaria; 25 Australian Prostate Cancer Research Centre-Qld, Institute of Health and Biomedical Innovation and School of Biomedical Science, Queensland University of Technology, Brisbane 4006, Australia; 26 Molecular Cancer Epidemiology Laboratory, Queensland Institute of Medical Research, Brisbane 4006, Australia; 27 Royal Marsden National Health Service (NHS) Foundation Trust, London and Sutton SM2 5PT, UK

**Keywords:** height, SNPs, gene and environment interaction, prostate cancer

## Abstract

**Background:**

Evidence on height and prostate cancer risk is mixed, however, recent studies with large data sets support a possible role for its association with the risk of aggressive prostate cancer.

**Methods:**

We analysed data from the PRACTICAL consortium consisting of 6207 prostate cancer cases and 6016 controls and a subset of high grade cases (2480 cases). We explored height, polymorphisms in genes related to growth processes as main effects and their possible interactions.

**Results:**

The results suggest that height is associated with high-grade prostate cancer risk. Men with height >180 cm are at a 22% increased risk as compared to men with height <173 cm (OR 1.22, 95% CI 1.01–1.48). Genetic variants in the growth pathway gene showed an association with prostate cancer risk. The aggregate scores of the selected variants identified a significantly increased risk of overall prostate cancer and high-grade prostate cancer by 13% and 15%, respectively, in the highest score group as compared to lowest score group.

**Conclusions:**

There was no evidence of gene-environment interaction between height and the selected candidate SNPs.

Our findings suggest a role of height in high-grade prostate cancer. The effect of genetic variants in the genes related to growth is seen in all cases and high-grade prostate cancer. There is no interaction between these two exposures.

Prostate cancer is the second most common cancer in men worldwide. Approximately 1.1 million men were diagnosed with prostate cancer in 2012 and almost 70% of the cases occur in more developed regions (IARC, 2014). The established risk factors include are age, ethnicity, family history, and over 100 common genetic variants. There are however other risk factors with less conclusive evidence including height (Key *et al*, 1997; Hayes *et al*, 1999; Villeneuve *et al*, 1999; Hsing *et al*, 2000; Norrish *et al*, 2000; Stattin *et al*, 2000). Height is a phenotypic trait determined by a combination of genetics and environmental factors. The relationship between height and prostate cancer risk has been proposed to act through possible factors including pre-adult nutritional status, androgen and insulin-like growth factor-I (IGF-I; Giovannucci *et al*, 1997; Calle, 2000; Willett, 2000; Freeman *et al*, 2001; Emerging Risk Factors Collaboration, 2012; Travis *et al*, 2016).

Height *per se* is not a cause of cancer but it is a marker for other exposures. It has also been suggested that taller stature may indicate increased risk of a number of cancers. The most consistent evidence has been found in relation to breast cancer (Willett, 2000; Gunnell *et al*, 2001).

In 2008, findings from a large nested case–control study (ProtecT) and meta-analysis (58 studies) suggested a positive association of height with high-grade prostate cancer (OR: 1.23; 95% CI: 1.06–1.43; Zuccolo *et al*, 2008). In this article, we present results from the international collaboration, the Prostate Cancer Association Group to Investigate Cancer Associated Alterations in the Genome consortium (PRACTCAL; http://practical.ccge.medschl.cam.ac.uk/). The aim was to explore the effects of height on prostate cancer risk. We were also interested to see if selected candidate SNPs related to height were associated with prostate cancer risk. Finally, we explored possible interactions between the selected SNPs and height.

## Materials and methods

### PRACTICAL consortium

The PRACTICAL consortium consists of 78 study groups around the world. The consortium was established in September 2008. The co-ordination of PRACTICAL is funded by Cancer Research UK and data have been contributed to the Collaborative Oncology Gene-environment Study (COGS), a project funded by the European Commission and 7th Framework Programme and the NIH grant. Each study with relevant data contributed an epidemiological data set and blood samples. Data on epidemiological factors for each study were provided in accordance with an assembled data dictionary. We performed quality control checks for each study before merging the data into one combined database. The majority of the samples are of European ancestry (95%). Since we investigated height as our main exposure we only analysed studies that contained subjects with European ancestry in order to minimise variation of height potentially influenced by different ethnic groups.

Blood derived DNA samples were genotyped for 211,155 SNPs on a custom Illumina array (iCOGS) in 25 074 prostate cancer cases and 24 272 controls. Details of genotyping and quality control analysis can be found in previous publication (Eeles *et al*, 2013).

### Analysis of height exposure

During the QC process, any subjects with outlier values were checked directly with the individual study group and subsequently either corrected or excluded. Height data were available in 10 out of 15 studies that submitted data on epidemiological factors. The inclusion criterion for this particular analysis is subjects with European ancestry. The total number of prostate cancer cases and controls were 6207 cases and 6016 controls. The list of studies included in the height exposure analyses are listed in Supplementary Table 1. Meta-analysis was performed using Meta-Analyst software (Wallace *et al*, 2009). We performed analysis in all PCA cases and high grade cases as compared to controls. The latter is defined by Gleason grade ⩾7. Out of 6207 cases, 2480 cases are high grade cases. Meta-analysis was carried out in 9 studies as one of the studies had no controls. Height was fitted as a continuous variable and study heterogeneity was explored. We also performed analysis whereby height was categorised into quartiles using control height values to determine the ranges. Results suggest study homogeneity hence results from a fixed effect model are reported. Pooled analysis was also performed. Tests for trend were carried out to assess possible dose-response relationships. Analyses were performed using IBM SPSS Statistics version 20.0. All analyses were adjusted for age, family history of prostate cancer, and study sites. As the data were derived from various studies with differing sample sizes, the analyses were therefore adjusted for study site to avoid possible confounding effects.

### SNPs analyses

We explored the effects of candidate SNPs related to growth factors on prostate cancer risk. We identified 168 candidate SNPs in *IGF-I*, *GH-1*, *SHOX*, *FMR1*, *GHITM*, and *GHRHR* genes related to human growth based on evidence from the literature and these SNPs were genotyped within a custom Illumina array (iCOGS). The full list of 168 candidate SNPs and associated relative risk estimates are shown in Supplementary Table 2. To evaluate effect sizes of these SNPs, we created a data set consisting of individual subjects whose IDs appeared in both the genotype and epidemiological data sets by matching the IDs between the two sets. We included only Caucasian subjects. This resulted in 13 123 controls and 9424 cases. PLINK software was used to explore minor allele frequency (MAF) and Hardy-Weinberg equilibrium (HWE; Purcell *et al*, 2007). MAF ranges were from 0.017 to 0.496. Out of 155 SNPs, 168 SNPs met HWE (*P*>0.05). STATA (version14) was used to obtain risk estimates and R-square (LDscore; Cheng *et al*, 2006). To quantify risk, the log-additive model was used by including a single variable coded as 0, 1, or 2 based additively on the number of minor alleles. Multiple logistic regression analyses were carried out to obtain the odd ratios of all 168 SNPs. Variables included in the model were age, family history of prostate cancer, study sites, principal components for European ancestry, and SNPs. Twelve SNPs showed significant associations (*P*-value <0.05). We then computed the R-square value for these 12 SNPs (Table 1). The results showed that these SNPs fell into 4 regions. SNPs were excluded if *r*^2^ value was >0.8 among them and we kept the most informative SNP based on association and *P*-value in each region. R-squared values for these 8 SNPs were less than 0.26. After this process, eight SNPs were selected for further analysis. Among these significant SNPs, only two yielded odds ratios (ORs) above 1.15.

### Gene and environment interaction analyses

We carried out gene and environment (GE) analyses in 6207 cases and 6016 controls. These are subjects with data on genotype and height. We applied two type of analyses based on the effect sizes of the SNP analyses.

1. For the 8 SNPs that were significantly associated with prostate cancer risk, individual standardised genetic score was computed. First, we multiplied coefficient for each SNP derived from multiple logistic regression (as explained above) with individual risk allele of that particular SNPs. To obtain total genetic risk score, we summed results from each SNP. To compute standardised score, the total score was divided with s.d. value from control group. First, genetic risk scores were analysed as for main effect by comparing subjects in the second and third tertile to the referent category. For GE analysis, both height and genetic risk score were then compared as binary variables. We classified both variables into tertiles with lowest tertile as reference group and highest tertiles as exposed group. We applied empirical-Bayes (EB) method proposed by Mukherjee *et al* (Mukherjee *et al*, 2008). Results for all PCA and high grade cases are presented.
2. We also employed the general multifactor dimensionality reduction (GMDR) method (Chen *et al*, 2011). For this we included the top 2 SNPs with effect sizes >1.15 and fitted these into the model at the same time. This procedure is not possible in the conventional GE methods. Height was fitted as a binary variable. We included subjects with height in the reference (lowest tertile) and top third tertile. Analyses were carried out for all PCA and high grade cases. Age and family history of PCA were fitted as covariates.

## Results

**Figure 1:**
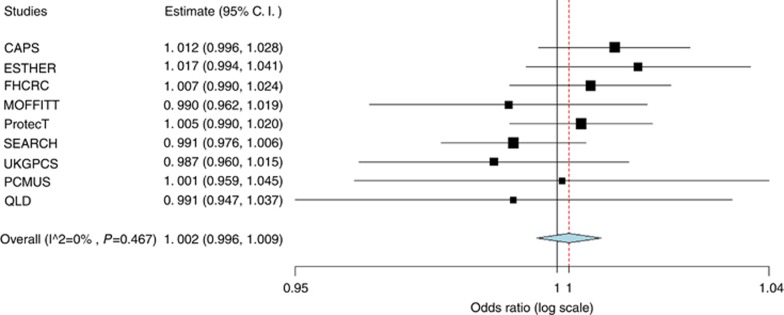
**Forest plot (all prostate cancer cases).**

**Figure 2:**
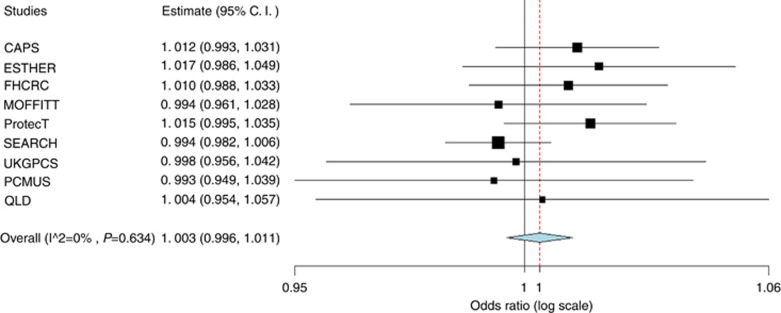
**Forest plot (high grade cases).**

Subject characteristics are displayed in Table 2. Family history of prostate cancer is associated with prostate cancer risk. Subjects with a positive family history of prostate cancer had a 12% increase in prostate cancer risk. Mean height for cases and controls was 176.3 and 176.8 cms, respectively. The Student’s *t*-test suggests a significant difference in the means between the two groups (*P*-value <0.05). Results from a meta-analysis of height are presented in Figures 1 and 2. ORs were adjusted for age, family history of prostate cancer, and study site. In all cases and high grade cases, point risk estimates of each study are very similar and are close to 1. None of the estimated relative risks is statistically significant. The heterogeneity *P*-value of 0.467 in all cases and 0.634 in high grade cases suggests that studies are homogenous. ORs of fixed effect model in all cases and high grade cases are 1.002 (95% CI 0.996–1.009) and 1.003 (95% CI 0.996–1.011) respectively.

Results from pooled analysis yielded similar risk estimates with OR 1.004, 95% CI 0.996–1.012 in all cases and OR 1.007, 95% CI 0.999–1.015 in high grade cases. We also analysed height as a categorical variable. Results are presented in Table 3. Results also suggest no overall association between height and prostate cancer risk comparing all cases with controls. In the high-grade case group, however, significant results were observed in the fourth quartile as compared to the first quartile (OR 1.22, 95% CI 1.014–1.477).

Table 4 shows the ORs of candidate SNPs with statistically significant results. ORs range from 0.90 to 1.32 with *P*-value from 10^−2^ to 10^−3^. One SNPs in the IGF-I gene had the highest ORs (1.32).

Table 5 shows the ORs of genetic risk scores and prostate cancer risk. A significant result was observed in the third tertile as compared to reference tertile (OR 1.13 with 95% CI 1.03–1.23) when all prostate cancer cases were included. The *P*-value for trend is also statistically significant. In the high grade cases, similar results were observed. There is also a trend of increasing risk with increasing genetic risk scores in all prostate cancer cases and in high grade cases.

The interaction results between height and genetic risk scores suggest that there is no GE interaction between height and genetic risk score (Table 6) regardless of type of cases.

Results of the GE analyses by GMDR method are depicted in Table 7. We fitted 2 SNPs with effect sizes >1.15 into the model and adjusted for covariates (age and family history of PCA). None of the models yield significant ORs regardless of case type. This is confirmed by cross-validation consistency. Both all and high grade cases, the extended models show consistency across testing sets.

## Discussion

This study investigated the effect of height and its possible interaction with selected SNPs from the PRACTICAL consortium in 6207 cases and 6016 controls. The consortium is an international collaboration on PCA and it has had notable successes for example in identifying 100 new genetic loci (Eeles *et al*, 2008, 2009, 2013; Al Olama *et al*, 2009, 2012, 2014). These loci confer small to medium risks with highly significant *P*-values of ⩽10^−7^ (GWAS significance).

There are, however, many polymorphisms with estimated risks less statistically significant which could still play an important role, particularly in the presence of environmental exposure. We therefore created a data set (subjects with epidemiological data and genotype data) which allowed us to investigate such a hypothesis.

Out of the 6207 cases, 2480 cases (40%) are high grade cases defined by Gleason grade ⩾7. One of the limitations of defining high grade cases is that we did not have data on Gleason grade breakdown hence we have to use combined score data of 7 rather than (4+3 or 3+4). Age and family history of PCA are confirmed risk factors in our study (Table 2). We investigated height in 3 ways. First, we explored height phenotype as a main exposure. Second, we investigated genetic profile (candidate SNPs) related to height, and third, we determined if there are any potential interactions between the selected SNPs and height. SNPs were deemed ‘related to height’ because they are found in candidate genes for height but they have not necessarily been identified in GWAS as underlying the variability of the height phenotype. We present results for all PCA cases and high grade cases as compared to controls. Although mean height values were very similar between cases and controls the mean difference was statistically significant and is in the opposite direction to that expected. In a multivariate analysis adjusted for age, family history of PCA and study sites, height as a continuous variable did not show associations with PCA risk in either all PCA cases or high grade PCA cases. However, height categorised in quartiles did show significantly increased risk in high grade cases. Subjects with a height >180 cm are at 22% increased risk compared with subjects with height <173 cm. We did not observe any association between height and low grade cases ((Gleason grade <7) results are not presented in the paper). Our findings suggest taller subjects are at increased risk of high grade PCA risk. A previous report from a large nested case–control study (ProtecT) reported the OR of prostate-specific antigen–detected high-grade PCA per 10 cm increase in height was 1.23; 95% CI: 1.06–1.43. In a meta-analysis of 58 studies, a smaller effect was reported (random-effects OR: 1.12; 95% CI: 1.05–1.19) (Zuccolo *et al*, 2008). Findings from The Early Stage Prostate Cancer Cohort Study which looked at the relationship between height and prostate cancer grade in various subpopulations of men with potentially different risk of high-grade PCA also suggested that participants in the highest quartile of height were more than twice as likely to have a Gleason score ⩾7 (4+3) at biopsy than participants in the lowest quartile of height (OR 2.14 (95% CI 1.11, 4.14); Farwell *et al*, 2011). Two other studies presented results exclusively on cases with advanced stage PCA and both supported a positive association between height and PCA risk (Hayes *et al*, 1999; Norrish *et al*, 2000). Hayes and colleagues observed a two-fold increased risk in white men with height>1.75 metres compared to height<1.67 metres. The association was absent among black men (Hayes *et al*, 1999). Norrish and colleagues investigated the role of height and PCA risk in both sporadic cancer cases and familial cancer cases. The study used the Gleason grading score to characterise the cases. Advanced PCA cases were defined by combined Gleason score ⩾7 and localised PCA cases by combined Gleason score ⩽6. Results on sporadic advanced cancer showed an indication of risk increasing across the quintiles (*p* for trend=0.07) which is similar to our high grade cases. Moreover the risk was greater among those with a positive family history of PCA (OR for height >179 cm compared to <170 cm=7.41, 95% CI 1.68–32.67, *p* for trend=0.02). A null association was reported in localised cases. Not only is height potentially associated with PCA risk but it also shows association with PCA mortality. A recent publication including more than 1 million subjects investigated adult height and the risk of cause-specific death and vascular morbidity suggested that hazard ratios per 6.5 cm greater height were 1.04 (1.03–1.06) for death from cancers and 1.07 (1.02–1.11) for death from PCA (Emerging Risk Factors Collaboration, 2012). In contrast, the results form a large cohort of 10 501 PCA cases and 10 831 controls within the NCI Breast and Prostate Cancer Cohort Consortium (BPC3) suggested that height was not associated with PCA risk both as a continuous variable (OR: 1.001, 95% CI: 1.000–1.002 per cm increase, *P*=0.12) or as in tertiles (OR: 1.02, 95% CI: 0.99–1.06, *P*=0.24) (Lindstrom *et al*, 2011). A null association was reported in the study also using PRACTICAL genotype data set and investigated the effect of height and prostate cancer incidence and mortality using Mendelian randomisation approach (Davies *et al*, 2015). The authors analysed genetic variants associated with height from published genome-wide association studies and reported that these genetic variants are strong instrument for the variable. There are some limitations in that GWA studies will not explain a majority of the estimated 80% contribution of genetic factors to variation in height (Lango Allen *et al*, 2010).

Human height is well known as a polygenic trait with a number of genes that contribute to height (Chial, 2008). Recent GWAS studies have identified strong and moderate effects of genes related to human height (Weedon and Frayling, 2008; McEvoy and Visscher, 2009). Single SNPs with small effects in aggregate form can be applied to assign individuals to their height distribution (Lettre, 2009). We applied a candidate SNPs approach and identified SNPs in genes that had been genotyped in our consortium that were related to growth processes. These SNPs were in the genes *IGF-I*, *GH-1*, *SHOX*, *FMR1*, *GHITM*, and *GHRHR* (Gunnell, 2000; Ellis *et al*, 2001; Gunnell *et al*, 2001). Twelve SNPs in these genes show significant associations. We computed *r*^*2*^ and kept the 8 SNP based on association and *P*-value in each region. Only one SNP (rs6503691) showed a small protective effect. This SNP is reported to associate with significantly decreased risk of breast cancer (Johansson *et al*, 2007; Zhao *et al*, 2015). Polymorphisms in the IGF signalling pathway have been shown to associate with PCA mortality (Cao *et al*, 2014). Other studies reported null associations (Gu *et al*, 2010; Tsilidis *et al*, 2013). We also explored association between aggregated SNPs score as main effect; results support that individuals with genetic risk scores in the third tertiles are at increased risk of high grade PCA at 15% and of all PCA cases at 13% as compared with the lowest tertile. A test for trend also supports a dose-response relationship (*P*-value <0.05 in both case groups). These findings support that a genetic risk score in the growth pathway are associated with high grade PCA. *IGF* genes have been previously linked with PCA (Cheng *et al*, 2006; Johansson *et al*, 2007; Cao *et al*, 2014; Gan *et al*, 2014; Qian *et al*, 2014; Takeuchi *et al*, 2014; Travis *et al*, 2016). *GHSR* genes are also previously reported to associate with prostate cancer risk (Dressen, 2007). We also investigated possible gene-environment interactions using two approaches. The first approach uses combined genetic risk scores and a binary variable of height with the first tertile as the reference group and the third tertile as the ‘exposed’ group. Analyses were done in both PCA and high grade case group using the Bayesian method proposed by (Mukherjee *et al*, 2008). Results of the GE analyses however suggested no interaction between genetic risk scores and height. In the second approach, we selected the top 2 SNPs with the strongest effect sizes and fitted a model using the GMDR method (Chen *et al*, 2011). The GMDR method allows adjustment for discrete and quantitative covariates and is applicable to both dichotomous and continuous phenotypes. The GMDR with covariate adjustment had a power of >80% in a case–control design with a sample size of ⩾2000. We applied the GMDR method because it differs from the traditional GE method in that it allows more than 1 SNP in the model (traditional method-based on the concept of single-factor–based approaches; Lou *et al*, 2007). The results also showed no interactions. None of the main effect (height) and extended models showed any significant results.

In summary, our findings suggest that height and genetic variants related to the human growth pathway are associated with high grade PCA risk. Taller men of >1.80 m are at increased risk of high grade PCA. Genetic variants in genes that relate to growth pathways are associated with prostate cancer risk. The estimated risk is evident amongst subjects in the highest score group when combined genetic risk scores were used. There is, however, no GE interaction between selected genetic variants and height.

## Figures and Tables

**Table 1:** *R*-square for 8 SNPs

**SNPs**	**rs11630647**	**rs11831436**	**rs13317803**	**rs2229765**	**rs2871864**	**rs35767**	**rs5742612**	**rs6503691**
rs11630647	1							
rs11831436	0.0000	1						
rs13317803	0.0001	0.0000	1					
rs2229765	0.0000	0.0001	0.0000	1				
rs2871864	0.2013	0.0001	0.0000	0.0030	1			
rs35767	0.0000	0.0484	0.0000	0.0000	0.0001	1		
rs5742612	0.0000	0.2629	0.0000	0.0000	0.0001	0.2080	1	
rs6503691	0.0002	0.0001	0.0001	0.0001	0.0000	0.0000	0.0001	1

Abbreviation: SNP=single-nucleotide polymorphism.

**Table 2:** Demographic data

				**95% CI**		
**Variables**	**Case**	**Control**	**OR**	**Lower**	**Upper**	***P*****-value**
**Age (years)**						
Number	6207	6016				
Mean±s.d.	63±7	60±7				<0.001^a^
**Family history of PCA**^b^						
No	4051	3594	1.00			
Yes	904	831	1.12	1.00	1.24	<0.05
**Height (cm)- all cases**						
Mean±s.d.	176.3±7.0	176.8±7.1				<0.001^a^
Number	2480	6016				
**Height (cm)- aggressive cases**						
Mean±s.d.	176.3±7.0	176.8±7.1				<0.05^a^

Abbreviations: CI=confidence interval; OR=odds ratio; PCA=prostate cancer.

a *P*-value of Student *t*-test.

b Adjusted for age.

**Table 3:** Height as quartiles and prostate cancer risk

		**All cases**					**High grade cases**			
			**95% CI**					**95% CI**		
**Height (cm)**	**Number of subjects** **(all cases+controls)**	**OR**^a^	**Lower**	**Upper**	***P*****-value**	**Number of subjects** **(High grade cases+controls)**	**OR**^a^	**Lower**	**Upper**	***P*****-value**
Q1 (⩽173.0)	2949	1.00				2000	1.00			
Q2 (173.1–177.9)	3210	1.16	0.99	1.35	0.064	2196	1.20	0.99	1.46	0.069
Q3 (178.0–180.0)	1865	1.07	0.89	1.28	0.467	1331	1.19	0.94	1.49	0.150
Q4 (>180.0)	4199	1.11	0.96	1.28	0.173	2969	1.22	1.01	1.48	0.035

Abbreviations: CI=confidence interval; OR=odds ratio.

All cases *P* for trend 0.407.

High-grade cases *P* for trend 0.075.

a Adjusted for age, family history and study sites.

**Table 4:** Candidate SNPs with significant associations

				**95% CI**		
**SNP**	**Minor allele**	**Genes**	**Odds ratios**^a^	**Lower**	**Upper**	***P*****-value**
rs6503691	A	*GHDC:STAT5B:STAT5A*	0.90	0.82	0.99	0.036
rs13317803	G	*GHSR:TNFSF10*	1.08	1.01	1.14	0.016
rs11831436	A	*IGF1*	1.19	1.01	1.41	0.040
rs35767	A	*IGF1*	1.12	1.03	1.22	0.006
rs5742612	G	*IGF1*	1.32	1.13	1.55	0.001
rs11630647	A	*IGF1R*	1.08	1.01	1.15	0.035
rs2871864	C	*IGF1R*	1.11	1.01	1.22	0.031
rs2229765	A	*IGF1R:PGPEP1L*	1.08	1.02	1.15	0.013

Abbreviations: CI=confidence interval; SNP=single-nucleotide polymorphism.

a Multiple logistic regression adjusted for age, family history of prostate cancer, study site and Principal Components of EU ancestry.

**Table 5:** Estimated risk of genetic risk scores (standardised score) and prostate cancer risk

	**All PCA**				**High grade PCA**			
		**95% CI**				**95% CI**		
**Genetic risk score**	**Odds ratio**^a^	**Lower**	**Upper**	***P*****-value**	**Odds ratio**^a^	**Lower**	**Upper**	***P*****-value**
Reference	1.00				1.00			
2nd tertile	1.06	0.97	1.16	0.186	1.03	0.92	1.16	>0.05
3rd tertile	1.13	1.04	1.23	0.006	1.55	1.03	1.29	<0.05

Abbreviations: CI=confidence interval; PCA=prostate cancer. All cases *P* for trend 0.006, high-grade cases *P* for trend 0.014.

a Adjusted for height.

**Table 6:** GE interaction result (Bayesian method)–interaction between Height and genetic risk scores

	***G*****=0**		***G*****=1**				**95% CI**	
**Group**	***E*****=0**	***E*****=1**	***E*****=0**	***E*****=1**	**Total**	**Estimated interaction OR**	**Lower**	**Upper**
Control	666	743	670	725	2804			
All PCA case	738	629	766	743	2876	1.14	0.98	1.33
High-grade PCA case	293	248	305	305	1151	1.18	0.94	1.49

Abbreviations: CI=confidence interval; PCA=prostate cancer.

*G*=0-subjects with genetic risk score in the first tertile, *G*=1-subjects with genetic risk score in the third tertile.

*E*=0-subjects with height in the first tertile, *E*=1-subjects with height in the third tertile.

**Table 7:** GE with 2 IGF-I pathway SNPs by GMDR method

**Group**	**Best model**	**Testing accuracy**	**Testing sensitivity**	**Testing odds ratio**^a^	**Testing** ***χ***^**2**^	**Cross-validation consistency**
All PCA cases	Height	0.51	0.51	1.07 (95% CI 0.72–1.59)	0.69 (*P*=0.408)	10/10
	Height, rs5742612	0.51	0.51	1.12 (95% CI 0.75–1.66)	0.84 (*P*=0.358)	10/10
	Height, rs5742612, rs11831436	0.51	0.51	1.09 (95% CI 0.73–1.61)	0.75 (*P*=0.387)	10/10
High grade cases	Height	0.51	0.54	1.16 (95% CI 0.63–2.11)	0.63 (*P*=0.429)	10/10
	Height, rs11831436	0.51	0.52	1.06 (95% CI 0.58–1.94)	0.22 (*P*=0.636)	10/10
	Height, rs5742612, rs11831436	0.51	0.53	1.12 (95% CI 0.62–2.05)	0.44 (*P*=0.507)	10/10

Abbreviations: GE=gene and environment; GMDR=general multifactor dimensionality reduction; IGF=insulin-like growth factor; PCA=prostate cancer; SNP=single-nucleotide polymorphism.

a Testing odds ratios adjusted for age and family history of prostate cancer.
