# Height, selected genetic markers and prostate cancer risk: results from the PRACTICAL consortium

**DOI:** 10.1038/bjc.2018.6

**Published:** 2018-02-13

**Authors:** Artitaya Lophatananon, Sarah Stewart-Brown, Zsofia Kote-Jarai, Ali Amin Al Olama, Sara Benlloch Garcia, David E Neal, Freddie C Hamdy, Jenny L Donovan, Graham G Giles, Liesel M Fitzgerald, Melissa C Southey, Paul Pharoah, Nora Pashayan, Henrik Gronberg, Fredrik Wiklund, Markus Aly, Janet L Stanford, Hermann Brenner, Aida K Dieffenbach, Volker Arndt, Jong Y Park, Hui-Yi Lin, Thomas Sellers, Chavdar Slavov, Radka Kaneva, Vanio Mitev, Jyotsna Batra, Amanda Spurdle, Judith A Clements, APCB BioResource, The PRACTICAL consortium, Douglas Easton, Rosalind A Eeles, Kenneth Muir

**Correction to**: *British Journal of Cancer* (2017) **117**, 734–743; doi:10.1038/bjc.2017.231; published online 01 August 2017

Since the publication of this paper, the authors have noticed that Sarah Stewart-Brown was linked to an incorrect affiliation. She should have been linked to affiliation 2, which is as follows:

Division of Health Sciences, Warwick Medical School, University of Warwick, Coventry CV4 7AL, UK

