# Supplementary material for: Height, selected genetic markers and prostate cancer risk: results from the PRACTICAL consortium
Source: Br J Cancer. 2017 Aug 1;117(5):734–43. doi: 10.1038/bjc.2017.231 (PMC5572182; doi:10.1038/bjc.2017.231)
Supplement: Supplementary Table 1 [file bjc2017231x1.docx]

**Supplement table1 List of studies including in height main exposure analysis**

| **Study description** | **Study design** | **Study acronym** | **Cases** | **Controls** | **Total** |
| --- | --- | --- | --- | --- | --- |
| Cancer of the Prostate in Sweden | Case-control | CAPS | 1126 | 648 | 1774 |
| Epidemiological investigations of the chances of preventing, recognizing early and optimally treating chronic diseases in an elderly population | population-based cohort study | ESTHER | 308 | 311 | 619 |
| Fred Hutchinson Cancer Research Center | Population-based case-control | FHCRC | 603 | 636 | 1239 |
| Melbourne Collaborative Cohort Study | population-based cohort study | MCCS | 622 | 0 | 622 |
| The Moffitt Group | Case-control | MOFFITT | 396 | 95 | 491 |
| Prostate testing for cancer and Treatment | Prospective | ProtecT | 1119 | 815 | 1934 |
| Study of Epidemiology and Risk factors in Cancer Heredity | Case-control | SEARCH | 895 | 1014 | 1909 |
| U.K. Genetic Prostate Cancer Study and The Prostate Cancer Research Foundation Study (now PCUK) | Case- control | UKGPCS | 836 | 2349 | 3185 |
| Prostate Cancer study Medical University Sofia | Case-control | PCMUS | 147 | 66 | 213 |
| Retrospective Queensland Study (QLD) and the Prostate Cancer Supportive Care and Patient Outcomes Project (ProsCan) | Case-control | QLD | 155 | 82 | 237 |
|  |  | Total | 6207 | 6016 | 12223 |
