# Supplementary material for: Height, selected genetic markers and prostate cancer risk: results from the PRACTICAL consortium
Source: Br J Cancer. 2017 Aug 1;117(5):734–43. doi: 10.1038/bjc.2017.231 (PMC5572182; doi:10.1038/bjc.2017.231)
Supplement: Supplementary Table 2 [file bjc2017231x2.docx]

**Supplementary table2 risk estimates of 168 candidate SNPs and prostate cancer risk**

| **Genes** | **Chr** | **SNP number** | **Base pair** | **Minor allele** | **OR*** | **95% C.I.** | | **P-value** |
| --- | --- | --- | --- | --- | --- | --- | --- | --- |
|  |  |  |  |  |  | **lower** | **upper** |  |
| *FMR1:FMR1NB* | 0 | rs5904820 | 146872477 | A | 1.03 | 0.98 | 1.09 | 0.299 |
| *FMR1-AS1:FMR1:FMR1NB* | 0 | rs5904817 | 146845982 | G | 1.02 | 0.97 | 1.06 | 0.469 |
| *FMR1NB* | 0 | rs764631 | 146895941 | A | 1.01 | 0.97 | 1.05 | 0.690 |
| *GHDC:STAT5B:STAT5A* | 17 | rs6503691 | 37647616 | A | 0.90 | 0.82 | 0.99 | 0.036 |
| *GHITM* | 10 | rs11200843 | 85843687 | A | 1.09 | 0.87 | 1.37 | 0.451 |
| *GHITM* | 10 | rs12774292 | 85843762 | G | 1.00 | 0.94 | 1.06 | 0.981 |
| *GHITM:C10orf99* | 10 | rs12764717 | 85882437 | G | 1.04 | 0.94 | 1.15 | 0.410 |
| *GHITM:C10orf99* | 10 | rs3814214 | 85888841 | A | 1.00 | 0.94 | 1.07 | 0.877 |
| *GHITM:C10orf99* | 10 | rs4400739 | 85879485 | A | 1.00 | 0.93 | 1.06 | 0.904 |
| *GHITM:C10orf99* | 10 | rs7071034 | 85882709 | G | 1.01 | 0.94 | 1.07 | 0.870 |
| *GHITM:C10orf99:CDHR1* | 10 | rs12412411 | 85896082 | T | 1.01 | 0.95 | 1.08 | 0.736 |
| *GHITM:C10orf99:CDHR1:LRIT2* | 10 | rs11200904 | 85923899 | A | 0.91 | 0.76 | 1.09 | 0.304 |
| *GHITM:C10orf99:CDHR1:LRIT2* | 10 | rs11594887 | 85926668 | A | 1.00 | 0.91 | 1.09 | 0.948 |
| *GHITM:C10orf99:CDHR1:LRIT2:LRIT1* | 10 | rs11597918 | 85937265 | A | 1.01 | 0.93 | 1.09 | 0.888 |
| *GHITM:C10orf99:CDHR1:LRIT2:LRIT1:RGR* | 10 | rs11200917 | 85948719 | G | 0.99 | 0.92 | 1.07 | 0.830 |
| *GHITM:C10orf99:CDHR1:LRIT2:LRIT1:RGR* | 10 | rs17103203 | 85948198 | A | 1.03 | 0.94 | 1.12 | 0.592 |
| *GHR* | 5 | rs10755268 | 42420104 | C | 1.04 | 0.96 | 1.12 | 0.363 |
| *GHR* | 5 | rs11744988 | 42510453 | A | 1.09 | 0.96 | 1.23 | 0.176 |
| *GHR* | 5 | rs11955075 | 42616517 | C | 1.01 | 0.94 | 1.08 | 0.877 |
| *GHR* | 5 | rs12153009 | 42589636 | A | 1.02 | 0.95 | 1.08 | 0.650 |
| *GHR* | 5 | rs12521020 | 42601600 | A | 0.99 | 0.92 | 1.06 | 0.729 |
| *GHR* | 5 | rs13153388 | 42543090 | C | 0.99 | 0.94 | 1.06 | 0.850 |
| *GHR* | 5 | rs13171720 | 42478472 | A | 0.94 | 0.87 | 1.01 | 0.098 |
| *GHR* | 5 | rs13184352 | 42533379 | A | 0.96 | 0.90 | 1.03 | 0.283 |
| *GHR* | 5 | rs13188386 | 42509312 | A | 0.98 | 0.91 | 1.05 | 0.525 |
| *GHR* | 5 | rs1509461 | 42443617 | G | 1.01 | 0.95 | 1.07 | 0.833 |
| *GHR* | 5 | rs28943882 | 42630868 | A | 0.95 | 0.88 | 1.02 | 0.163 |
| *GHR* | 5 | rs2940930 | 42441268 | A | 1.03 | 0.97 | 1.09 | 0.389 |
| *GHR* | 5 | rs2940944 | 42524899 | C | 0.99 | 0.94 | 1.06 | 0.864 |
| *GHR* | 5 | rs2972400 | 42461587 | A | 1.03 | 0.96 | 1.12 | 0.396 |
| *GHR* | 5 | rs2972418 | 42519450 | G | 1.01 | 0.95 | 1.07 | 0.747 |
| *GHR* | 5 | rs2972419 | 42519634 | A | 1.04 | 0.96 | 1.12 | 0.323 |
| *GHR* | 5 | rs33939197 | 42531815 | G | 0.93 | 0.87 | 1.00 | 0.057 |
| *GHR* | 5 | rs4130113 | 42550408 | A | 1.01 | 0.95 | 1.07 | 0.837 |
| *GHR* | 5 | rs4395642 | 42588188 | A | 1.00 | 0.93 | 1.07 | 0.992 |
| *GHR* | 5 | rs4610467 | 42559225 | A | 0.94 | 0.79 | 1.12 | 0.471 |
| *GHR* | 5 | rs4866941 | 42653249 | A | 0.97 | 0.91 | 1.04 | 0.407 |
| *GHR* | 5 | rs6179 | 42735801 | A | 0.97 | 0.91 | 1.04 | 0.378 |
| *GHR* | 5 | rs6873545 | 42667021 | G | 0.97 | 0.91 | 1.04 | 0.399 |
| *GHR* | 5 | rs6878512 | 42703628 | A | 0.99 | 0.92 | 1.08 | 0.888 |
| *GHR* | 5 | rs6887528 | 42659309 | A | 1.03 | 0.91 | 1.16 | 0.679 |
| *GHR* | 5 | rs6897530 | 42703694 | G | 0.98 | 0.92 | 1.05 | 0.593 |
| *GHR* | 5 | rs7701605 | 42686227 | C | 0.99 | 0.92 | 1.06 | 0.720 |
| *GHR* | 5 | rs7712701 | 42605947 | A | 1.00 | 0.94 | 1.07 | 0.915 |
| *GHR* | 5 | rs7721081 | 42686141 | A | 0.99 | 0.92 | 1.08 | 0.854 |
| *GHR* | 5 | rs7732059 | 42459229 | C | 0.98 | 0.92 | 1.05 | 0.623 |
| *GHR* | 5 | rs7735889 | 42597956 | G | 0.99 | 0.92 | 1.06 | 0.741 |
| *GHR* | 5 | rs7736209 | 42613316 | A | 1.00 | 0.94 | 1.07 | 0.938 |
| *GHR:CCDC152* | 5 | rs6180 | 42754996 | C | 1.00 | 0.94 | 1.06 | 0.910 |
| *GHRHR* | 7 | rs12537375 | 30987987 | G | 1.02 | 0.93 | 1.12 | 0.690 |
| *GHRHR:ADCYAP1R1* | 7 | rs6462246 | 31016476 | A | 1.06 | 0.97 | 1.15 | 0.173 |
| *GHRHR:ADCYAP1R1* | 7 | rs7796157 | 31019584 | A | 1.00 | 0.91 | 1.11 | 0.937 |
| *GHRL:GHRLOS:SEC13:ATP2B2* | 3 | rs241508 | 10358996 | A | 0.97 | 0.91 | 1.03 | 0.351 |
| *GHSR:TNFSF10* | 3 | rs10513702 | 173669647 | A | 1.05 | 0.99 | 1.12 | 0.084 |
| *GHSR:TNFSF10* | 3 | rs13317803 | 173660458 | G | 1.08 | 1.01 | 1.14 | 0.016 |
| *GHSR:TNFSF10* | 3 | rs2100143 | 173685570 | G | 0.97 | 0.92 | 1.03 | 0.360 |
| *GHSR:TNFSF10* | 3 | rs6445063 | 173676326 | G | 0.92 | 0.84 | 1.00 | 0.057 |
| *GHSR:TNFSF10* | 3 | rs6777318 | 173688644 | A | 0.99 | 0.92 | 1.05 | 0.654 |
| *GHSR:TNFSF10* | 3 | rs9881073 | 173666094 | A | 1.07 | 1.01 | 1.13 | 0.031 |
| *GHSR:TNFSF10* | 3 | rs9881097 | 173668070 | A | 1.07 | 1.00 | 1.13 | 0.035 |
| *IGF1* | 12 | rs1019731 | 101388555 | A | 1.00 | 0.92 | 1.09 | 0.981 |
| *IGF1* | 12 | rs10735380 | 101368366 | G | 1.01 | 0.95 | 1.08 | 0.709 |
| *IGF1* | 12 | rs10745940 | 101321326 | A | 1.05 | 0.98 | 1.12 | 0.164 |
| *IGF1* | 12 | rs10778176 | 101387109 | A | 1.02 | 0.96 | 1.09 | 0.500 |
| *IGF1* | 12 | rs10860860 | 101304963 | A | 0.97 | 0.91 | 1.03 | 0.294 |
| *IGF1* | 12 | rs10860861 | 101309699 | G | 1.00 | 0.94 | 1.06 | 0.903 |
| *IGF1* | 12 | rs10860862 | 101310202 | A | 1.03 | 0.95 | 1.11 | 0.441 |
| *IGF1* | 12 | rs10860869 | 101389182 | T | 1.02 | 0.96 | 1.09 | 0.500 |
| *IGF1* | 12 | rs11111262 | 101322307 | A | 1.04 | 0.94 | 1.15 | 0.472 |
| *IGF1* | 12 | rs11111272 | 101351571 | C | 1.04 | 0.98 | 1.12 | 0.188 |
| *IGF1* | 12 | rs11111293 | 101445426 | G | 1.01 | 0.94 | 1.09 | 0.728 |
| *IGF1* | 12 | rs11831436 | 101377373 | A | 1.19 | 1.01 | 1.41 | 0.040 |
| *IGF1* | 12 | rs12821878 | 101391797 | A | 0.96 | 0.89 | 1.03 | 0.216 |
| *IGF1* | 12 | rs12833855 | 101352056 | A | 1.03 | 0.96 | 1.10 | 0.360 |
| *IGF1* | 12 | rs1520220 | 101320652 | G | 1.07 | 0.99 | 1.16 | 0.089 |
| *IGF1* | 12 | rs1549593 | 101320921 | A | 1.01 | 0.93 | 1.09 | 0.886 |
| *IGF1* | 12 | rs17727841 | 101333760 | G | 1.00 | 0.93 | 1.08 | 0.991 |
| *IGF1* | 12 | rs17797047 | 101429842 | C | 0.97 | 0.88 | 1.08 | 0.630 |
| *IGF1* | 12 | rs17797222 | 101438076 | G | 1.00 | 0.93 | 1.08 | 0.971 |
| *IGF1* | 12 | rs2033178 | 101371206 | A | 1.09 | 0.96 | 1.23 | 0.185 |
| *IGF1* | 12 | rs2139573 | 101302904 | G | 1.00 | 0.94 | 1.06 | 0.939 |
| *IGF1* | 12 | rs2162679 | 101395389 | G | 1.12 | 1.03 | 1.22 | 0.007 |
| *IGF1* | 12 | rs2288378 | 101354138 | A | 1.02 | 0.96 | 1.10 | 0.489 |
| *IGF1* | 12 | rs28399924 | 101320154 | A | 0.98 | 0.90 | 1.06 | 0.544 |
| *IGF1* | 12 | rs2946834 | 101311944 | A | 1.03 | 0.96 | 1.10 | 0.394 |
| *IGF1* | 12 | rs2971575 | 101305009 | G | 1.01 | 0.95 | 1.07 | 0.839 |
| *IGF1* | 12 | rs35765 | 101405826 | A | 1.02 | 0.93 | 1.12 | 0.662 |
| *IGF1* | 12 | rs35766 | 101404603 | G | 1.10 | 1.01 | 1.19 | 0.026 |
| *IGF1* | 12 | rs35767 | 101399699 | A | 1.12 | 1.03 | 1.22 | 0.006 |
| *IGF1* | 12 | rs4764697 | 101377302 | A | 1.01 | 0.95 | 1.08 | 0.781 |
| *IGF1* | 12 | rs4764883 | 101330435 | G | 1.05 | 0.98 | 1.12 | 0.147 |
| *IGF1* | 12 | rs5742612 | 101398994 | G | 1.32 | 1.13 | 1.55 | 0.001 |
| *IGF1* | 12 | rs5742620 | 101393730 | A | 1.16 | 0.97 | 1.38 | 0.102 |
| *IGF1* | 12 | rs5742629 | 101381393 | G | 1.03 | 0.96 | 1.10 | 0.438 |
| *IGF1* | 12 | rs5742632 | 101380604 | G | 1.00 | 0.93 | 1.07 | 0.948 |
| *IGF1* | 12 | rs5742652 | 101360196 | A | 1.13 | 0.93 | 1.38 | 0.213 |
| *IGF1* | 12 | rs5742665 | 101347680 | C | 0.95 | 0.87 | 1.04 | 0.244 |
| *IGF1* | 12 | rs5742671 | 101339888 | A | 1.00 | 0.93 | 1.08 | 0.973 |
| *IGF1* | 12 | rs5742678 | 101338462 | G | 1.03 | 0.97 | 1.10 | 0.355 |
| *IGF1* | 12 | rs5742692 | 101323728 | G | 1.14 | 0.90 | 1.45 | 0.276 |
| *IGF1* | 12 | rs5742714 | 101313982 | G | 1.04 | 0.94 | 1.15 | 0.469 |
| *IGF1* | 12 | rs6214 | 101317699 | A | 0.98 | 0.92 | 1.04 | 0.527 |
| *IGF1* | 12 | rs6219 | 101314322 | A | 1.04 | 0.94 | 1.15 | 0.470 |
| *IGF1* | 12 | rs6220 | 101318645 | G | 1.05 | 0.99 | 1.13 | 0.116 |
| *IGF1* | 12 | rs6539035 | 101325065 | G | 1.02 | 0.96 | 1.10 | 0.494 |
| *IGF1* | 12 | rs7136446 | 101362645 | G | 1.00 | 0.94 | 1.06 | 0.965 |
| *IGF1* | 12 | rs7956547 | 101382946 | G | 1.01 | 0.95 | 1.09 | 0.674 |
| *IGF1* | 12 | rs7971494 | 101378397 | A | 0.94 | 0.80 | 1.11 | 0.468 |
| *IGF1* | 12 | rs855211 | 101434940 | A | 1.08 | 1.00 | 1.17 | 0.056 |
| *IGF1* | 12 | rs9651925 | 101369674 | C | 1.01 | 0.95 | 1.08 | 0.705 |
| *IGF1* | 12 | rs972936 | 101349051 | A | 1.03 | 0.96 | 1.10 | 0.359 |
| *IGF1* | 12 | rs978458 | 101326369 | A | 1.03 | 0.97 | 1.11 | 0.326 |
| *IGF1* | 12 | rs9989002 | 101374353 | A | 1.02 | 0.96 | 1.09 | 0.513 |
| *IGF1R* | 15 | rs10794486 | 97119058 | G | 0.97 | 0.91 | 1.04 | 0.375 |
| *IGF1R* | 15 | rs11247367 | 97053799 | A | 1.11 | 0.98 | 1.26 | 0.087 |
| *IGF1R* | 15 | rs11247378 | 97231968 | A | 1.03 | 0.91 | 1.18 | 0.635 |
| *IGF1R* | 15 | rs11630647 | 97058470 | A | 1.08 | 1.01 | 1.15 | 0.035 |
| *IGF1R* | 15 | rs11632057 | 97059247 | A | 0.92 | 0.81 | 1.06 | 0.255 |
| *IGF1R* | 15 | rs11856426 | 96992523 | G | 0.97 | 0.92 | 1.03 | 0.361 |
| *IGF1R* | 15 | rs12050484 | 97172805 | A | 1.00 | 0.94 | 1.06 | 0.886 |
| *IGF1R* | 15 | rs12908437 | 97104898 | A | 0.97 | 0.91 | 1.03 | 0.327 |
| *IGF1R* | 15 | rs12910200 | 97262992 | A | 1.05 | 0.98 | 1.12 | 0.138 |
| *IGF1R* | 15 | rs1319869 | 97030008 | C | 0.98 | 0.89 | 1.08 | 0.663 |
| *IGF1R* | 15 | rs13329348 | 97021743 | G | 1.00 | 0.88 | 1.12 | 0.940 |
| *IGF1R* | 15 | rs13329408 | 97023676 | A | 1.03 | 0.95 | 1.12 | 0.524 |
| *IGF1R* | 15 | rs1357112 | 97250899 | G | 1.06 | 1.00 | 1.13 | 0.054 |
| *IGF1R* | 15 | rs1513643 | 97144284 | G | 0.98 | 0.93 | 1.04 | 0.576 |
| *IGF1R* | 15 | rs1521481 | 97231946 | C | 1.00 | 0.94 | 1.06 | 0.929 |
| *IGF1R* | 15 | rs1962589 | 97154292 | G | 1.04 | 0.95 | 1.14 | 0.385 |
| *IGF1R* | 15 | rs2175795 | 97130377 | A | 0.98 | 0.91 | 1.04 | 0.487 |
| *IGF1R* | 15 | rs2684761 | 97181893 | A | 0.98 | 0.92 | 1.04 | 0.444 |
| *IGF1R* | 15 | rs2684781 | 97227071 | G | 1.00 | 0.93 | 1.07 | 0.919 |
| *IGF1R* | 15 | rs2715417 | 97270570 | A | 0.96 | 0.91 | 1.02 | 0.226 |
| *IGF1R* | 15 | rs2871864 | 96979816 | C | 1.11 | 1.01 | 1.22 | 0.031 |
| *IGF1R* | 15 | rs3743259 | 97260684 | G | 1.00 | 0.94 | 1.07 | 0.913 |
| *IGF1R* | 15 | rs3743260 | 97260815 | A | 1.11 | 0.91 | 1.35 | 0.292 |
| *IGF1R* | 15 | rs3784606 | 97276004 | G | 0.94 | 0.88 | 1.01 | 0.110 |
| *IGF1R* | 15 | rs4305005 | 97032621 | G | 0.95 | 0.86 | 1.05 | 0.300 |
| *IGF1R* | 15 | rs4965425 | 96999186 | A | 0.98 | 0.93 | 1.04 | 0.578 |
| *IGF1R* | 15 | rs4966013 | 97062907 | G | 1.06 | 1.00 | 1.13 | 0.062 |
| *IGF1R* | 15 | rs6598534 | 96960825 | A | 1.00 | 0.94 | 1.06 | 0.964 |
| *IGF1R* | 15 | rs7168671 | 97272485 | A | 0.98 | 0.90 | 1.06 | 0.567 |
| *IGF1R* | 15 | rs7169544 | 97275275 | A | 1.02 | 0.96 | 1.09 | 0.439 |
| *IGF1R* | 15 | rs7403473 | 96983696 | G | 0.99 | 0.93 | 1.06 | 0.847 |
| *IGF1R* | 15 | rs8027767 | 97115026 | C | 0.98 | 0.92 | 1.04 | 0.515 |
| *IGF1R* | 15 | rs8030950 | 97221544 | A | 1.00 | 0.93 | 1.06 | 0.893 |
| *IGF1R* | 15 | rs8037002 | 97263686 | C | 1.02 | 0.96 | 1.08 | 0.576 |
| *IGF1R* | 15 | rs8041953 | 97179824 | G | 1.03 | 0.94 | 1.13 | 0.518 |
| *IGF1R* | 15 | rs867431 | 97267206 | G | 0.99 | 0.93 | 1.05 | 0.769 |
| *IGF1R* | 15 | rs883149 | 97267224 | T | 1.00 | 0.93 | 1.07 | 0.924 |
| *IGF1R:PGPEP1L* | 15 | rs12440962 | 97317587 | A | 1.00 | 0.94 | 1.08 | 0.928 |
| *IGF1R:PGPEP1L* | 15 | rs2229765 | 97295748 | A | 1.08 | 1.02 | 1.15 | 0.013 |
| *IGF1R:PGPEP1L* | 15 | rs2593053 | 97317779 | A | 0.98 | 0.92 | 1.04 | 0.561 |
| *IGF1R:PGPEP1L* | 15 | rs2654976 | 97367760 | G | 1.00 | 0.93 | 1.08 | 0.928 |
| *IGF1R:PGPEP1L* | 15 | rs2684787 | 97322585 | A | 1.01 | 0.95 | 1.09 | 0.707 |
| *IGF1R:PGPEP1L* | 15 | rs2684805 | 97283608 | G | 0.96 | 0.87 | 1.07 | 0.480 |
| *IGF1R:PGPEP1L* | 15 | rs28674628 | 97323351 | G | 1.03 | 0.83 | 1.27 | 0.813 |
| *IGF1R:PGPEP1L* | 15 | rs3743253 | 97317597 | A | 1.02 | 0.95 | 1.10 | 0.626 |
| *IGF1R:PGPEP1L* | 15 | rs3743262 | 97282996 | A | 0.96 | 0.83 | 1.11 | 0.600 |
| *IGF1R:PGPEP1L* | 15 | rs702497 | 97319104 | A | 1.01 | 0.94 | 1.08 | 0.858 |
| *IGF1R:PGPEP1L* | 15 | rs7167580 | 97312731 | A | 1.01 | 0.95 | 1.09 | 0.691 |
| *IGF1R:PGPEP1L* | 15 | rs8024849 | 97368405 | G | 1.01 | 0.93 | 1.10 | 0.829 |
| *IGF1R:PGPEP1L* | 15 | rs9672254 | 97315608 | A | 1.03 | 0.97 | 1.09 | 0.390 |
| *IGF1R:PGPEP1L* | 15 | rs9920651 | 97280163 | A | 1.07 | 0.99 | 1.15 | 0.073 |
| *SHOX2* | 3 | rs3107582 | 159260534 | A | 1.01 | 0.92 | 1.10 | 0.901 |
| *SHOX2:RSRC1* | 3 | rs1608114 | 159298074 | G | 1.01 | 0.93 | 1.10 | 0.823 |
| *SHOX2:RSRC1* | 3 | rs2686543 | 159265913 | A | 1.00 | 0.92 | 1.09 | 0.981 |
| *SHOX2:RSRC1* | 3 | rs6803630 | 159319759 | A | 1.01 | 0.95 | 1.07 | 0.838 |

Chr-chromosome, OR-odds ratio

* adjusted for age, family history of prostate cancer, study sites, Principal Components for European ancestry
